# Prevalence and incidence of Parkinson’s disease and drug-induced parkinsonism in Korea

**DOI:** 10.1186/s12889-019-7664-6

**Published:** 2019-10-22

**Authors:** Sola Han, Siin Kim, Hyungtae Kim, Hae-Won Shin, Kyoung-Sae Na, Hae Sun Suh

**Affiliations:** 1 College of Pharmacy, Pusan National University, Busan, South Korea; 2 Department of Neurology, Chung-Ang University College of Medicine, Seoul, South Korea; 3 Gachon University School of Medicine, Incheon, South Korea; 4 Pharmaceutical Economics, Outcomes Research & Policy, College of Pharmacy, Pusan National University, 2, Busandaehak-ro 63 beon-gil, Geumjeong-gu, Busan, 46241 South Korea

**Keywords:** Drug-induced parkinsonism, Parkinson’s disease, Prevalence, Incidence, Pharmacoepidemiology

## Abstract

**Background:**

Parkinson’s disease (PD) and drug-induced parkinsonism (DIP) are the major diseases of parkinsonism. To better understand parkinsonism, we aimed to assess the prevalence and incidence of PD and DIP in Korea from 2012 to 2015.

**Methods:**

We used the Health Insurance Review and Assessment Service database, which covers the entire population in Korea. We used claims during 2011–2015 to assess epidemiology of PD and DIP during 2012–2015. Retrospective cross-sectional study design was employed to assess prevalence, whereas retrospective cohort study design was used to determine incidence. Patients with at least one claim with ICD-10 G20 and who received antiparkinsonian drugs for at least 60 days were classified as having PD. We excluded patients with antiparkinsonian drugs that can be used for indications other than PD. Patients with at least one claim with ICD-10 G211 or G251 during the prescription period of drugs that are frequently related with DIP were classified as having DIP. Incident cases had a disease-free period of 1 year before diagnosis. To evaluate the significance of changes in the prevalence or incidence over time, Poisson regression was used to determine *p* for trend.

**Results:**

The prevalence of PD increased from 156.9 per 100,000 persons in 2012 to 181.3 per 100,000 persons in 2015 (*p* for trend< 0.0001). The incidence of PD decreased steadily from 35.4 per 100,000 person-years in 2012 to 33.3 per 100,000 person-years in 2015 (*p* for trend< 0.0001). The prevalence of DIP increased from 7.3 per 100,000 persons in 2012 to 15.4 per 100,000 persons in 2015 (*p* for trend< 0.0001) and the incidence of DIP increased from 7.1 per 100,000 person-years in 2012 to 13.9 per 100,000 person-years in 2015 (*p* for trend< 0.0001).

**Conclusions:**

Our study suggests that the incidence of PD has gradually decreased whereas, the incidence of DIP increased from 2012 to 2015. Further studies are warranted to examine possible causes of increased DIP incidence in order to develop management strategy for parkinsonism.

## Background

Parkinson’s disease (PD) is a common progressive neurodegenerative disorder that increases with age, with a reported prevalence of 315 per 100,000 persons of all ages [1]. According to a previous systematic review, a lower prevalence of PD is observed in all age groups in Asia than in other continents [1]. Drug-induced parkinsonism (DIP) has been recognized as the second most common form of parkinsonism after idiopathic Parkinson’s disease [2]. For several decades, drugs that can induce parkinsonism have been reported. These offending drugs include antipsychotics, calcium channel blockers, and gastrointestinal motility drugs [2]. Although there are no published large-scale epidemiological data, a recently published study that used data from the Olmsted county demonstrated that the incidence of DIP is 3.3 per 100,000 person-years in all age groups [3].

Considering the epidemiological findings [1, 3] of both PD and DIP, which show an increasing trend with age, and the fact that the aging population of many countries is increasing, more precise and up-to-date estimates of the prevalence and incidence of these disorders are needed to optimize the strategies for prevention and management [1, 3].

We conducted this population-based study to estimate the prevalence and incidence of PD and DIP, as well as the prevalence of the use of drugs that have been frequently related with DIP, in Korea from 2012 to 2015 using a large national claims database. The objective of this study was not simply to compare the incidence and prevalence of PD and DIP, but to assess the epidemiology of these diseases in a large-scale study which could help better understanding of parkinsonism because PD and DIP are the major diseases of parkinsonism.

## Methods

### Data source

This study used the Korean Health Insurance Review and Assessment (HIRA) Service database from 2011 to 2015, which is the data of the universal health insurance system in South Korea that covers the entire population [4]. If patients are diagnosed with PD or DIP, all of these patients are included in this database. This database contains patients’ demographics and the medical and pharmacy claims of approximately 50 million Koreans [4]. Diagnoses in this database were coded according to the Korean Standard Classification of Diseases (KCD), which is based on the *International Classification of Diseases 10th Revision* (ICD-10) [4]. HIRA provided the data after de-identification.

### Study population

Patients with PD from 2012 to 2015 were identified if they met all of the following criteria: (1) at least one claim with all of the available diagnosis codes (i.e., primary and all sub-diagnosis codes) of PD (ICD-10 code: G20); and (2) at least 60 days of supply of antiparkinsonian drugs prescribed by neurologists. The definition of PD was based on the expert opinions of Korean neurologists and previous Korean research published in 2007 [5].

Among all antiparkinsonian drugs [6], only antiparkinsonian drugs that are specifically used for the treatment of PD were included, based on the expert opinions of Korean neurologist. These include entacapone, levodopa combinations, pergolide, pramipexole, rasagiline, ropinirole, and selegiline (Additional file 1 Table S1). Other antiparkinsonian drugs, which were available in Korea during the analysis period, such as amantadine, apomorphine, benzatropine, biperiden, bromocriptine, cabergoline, dihydroergocryptine, orphenadrine, piribedil, procyclidine, rotigotine, and trihexyphenidyl, were excluded from analysis as these drugs can be used for indications other than PD.

Patients with DIP from 2012 to 2015 were identified if they met all of the following criteria: (1) at least one prescription for a drug that is known to be frequently related with DIP (i.e., offending drug); (2) the date of earliest claim with primary or secondary diagnosis code of DIP (ICD-10 code: G21.1, G25.1) occurred during the prescription period of the offending drug. Offending drugs include antiemetics (levosulpiride and metoclopramide), atypical antipsychotics (risperidone, olanzapine, and aripiprazole), typical antipsychotics (haloperidol, perphenazine, and chlorpromazine), and calcium channel blockers (flunarizine). The definition of DIP and the drugs that are known to be frequently related with DIP were based on the expert opinions of Korean neurologists and the published literature [2, 7].

A patient with PD or DIP was considered to be incident if it was preceded by a 12-month disease-free period; otherwise, it was considered to be prevalent.

### Statistical analysis

We estimated prevalences in a retrospective cross-sectional study design, whereas incidences were estimated based on the retrospective cohort study design. To estimate prevalences, we counted the number of patients with disease within a certain time frame in a cross-sectional way instead of following up patients. On the contrary, to estimate incidences, new cases have to be identified, thus we looked back a certain period of time to examine whether these patients are newly diagnosed without prior history. Prevalence was calculated as the number of cases of the disease divided by all population and expressed as number per 100,000 persons. Incidence density was calculated as the number of new cases of the disease divided by the person-year and expressed as number per 100,000 person-years. Age- and sex-specific annual prevalence and incidence density of PD and DIP in each of the 4 years (from 2012 to 2015) were calculated using denominators derived from mid-year Korean population of the respective stratum [8]. Because, as mentioned above, HIRA database covers the entire Korean population, numerator of prevalence and incidence were also derived from entire Korean population. In addition, we calculated the average prevalence and incidence over 2012–2015. For international comparison, age-standardized rates were calculated using direct standardization with age distribution of the US 2000 and WHO 2000–2025 population as reference populations [9, 10]. The female-to-male ratio for PD and DIP was also calculated. The differences in the prevalence and incidence density of PD and DIP between sexes were analyzed using the chi-square test [11]. To evaluate the significance of changes in the prevalence or incidence of PD and DIP over time, Poisson regression adjusted by sex was used [12] and a test for trend (i.e., the *P-*value for whether the coefficient for calendar year was significantly different from 0) was performed. All analyses were performed using SAS version 9.4 (SAS Inc., Cary, NC, USA). A *P*-value of less than 0.05 was considered to be statistically significant.

### Sensitivity analyses

First, to evaluate whether the prevalence and incidence of PD were influenced by the definition of PD, we assessed the prevalence and incidence of PD by restricting patients who met all of the following criteria to exclude patients with multiple system atrophy, DIP, or progressive supranuclear palsy: (1) had a primary diagnosis of PD without any secondary diagnosis of cerebellar ataxia (ICD-10 code: G111, G112, G113, G119, G312, R270), DIP (ICD-10 code: G211, G251), and Steele-Richardson-Olszewski syndrome (ICD-10 code: G231); and (2) received at least 60 days of supply of antiparkinsonian drugs at the Department of Neurology. This definition was based on the expert opinions of Korean neurologists.

Second, to assess whether patterns of prevalence or incidence of DIP changed after excluding antipsychotics, we assessed the prevalence and incidence of DIP by not including antipsychotics in the analysis. Antipsychotics were reported as the major causes of DIP in published literature [3].

## Results

### Prevalence of PD and DIP

The average prevalence of PD between 2012 and 2015 was 171.01 per 100,000 persons. The average age-standardized prevalence of PD according to the WHO 2000–2025 and US 2000 standard population between 2012 and 2015 was 114.13 and 176.21 per 100,000 persons, respectively. In contrast, the average prevalence of DIP between 2012 and 2015 was 9.78 per 100,000 persons and the average age-standardized prevalence of DIP according to the WHO 2000–2025 and US 2000 standard population between 2012 and 2015 was 7.99 and 8.95 per 100,000 persons, respectively (Table 1).

**Table 1:** The average prevalence and incidence of Parkinson’s disease and drug-induced parkinsonism in Korea between 2012 and 2015

	Prevalence ^a^				Incidence ^a^			
	Male	Female	Total	Female-to-Male Ratio	Male	Female	Total	Female-to-Male Ratio
Parkinson’s disease								
*Age group (years)*								
0–19	0.20	0.21	0.20	1.06	0.09	0.11	0.10	1.44
20–24	1.14	1.09	1.12	0.95	0.36	0.50	0.43	1.43
25–29	1.82	1.35	1.59	0.76	0.66	0.43	0.55	0.72
30–34	3.59	3.02	3.31	0.85	1.11	1.09	1.10	1.03
35–39	7.26	5.50	6.39	0.76*	2.09	1.63	1.87	0.78
40–44	13.33	10.68	12.03	0.80*	3.65	3.17	3.41	0.88
45–49	30.37	27.68	29.05	0.91	7.29	7.03	7.16	0.96
50–54	67.17	65.26	66.22	0.97	15.60	14.87	15.24	0.95
55–59	136.22	137.60	136.92	1.01	29.64	29.79	29.72	1.01
60–64	275.18	303.45	289.59	1.10*	59.55	62.35	60.98	1.05
65–69	554.05	596.42	576.41	1.08*	120.28	118.07	119.13	0.98
70–74	1017.86	1176.07	1106.37	1.16*	222.20	233.59	228.58	1.05
75–79	1565.52	1704.63	1649.67	1.09*	335.35	327.22	330.40	0.98
80-	1721.25	1434.80	1519.63	0.83*	373.14	253.97	289.30	0.68*
Total	139.58	202.43	171.01	1.45*	30.44	39.49	34.97	1.30*
*Age-standardized rates*								
WHO 2000–2025	113.10	116.00	114.13	–	24.75	22.97	23.49	–
US 2000	176.86	177.96	176.21	–	38.57	34.64	35.79	–
Drug-induced parkinsonism								
*Age group (years)*								
0–19	2.42	1.41	1.94	0.61*	2.30	1.34	1.84	0.60*
20–24	6.28	5.90	6.10	0.91	5.69	5.43	5.57	0.93
25–29	7.50	7.18	7.35	0.98	6.89	6.47	6.69	0.96
30–34	8.03	8.39	8.21	1.05	7.13	7.49	7.31	1.04
35–39	11.73	11.87	11.80	1.02	10.25	10.45	10.35	1.04
40–44	14.98	13.84	14.42	0.94	12.62	12.31	12.47	0.98
45–49	14.96	15.37	15.16	1.02	12.75	13.61	13.18	1.07
50–54	12.05	15.34	13.68	1.33*	10.64	13.36	11.99	1.31*
55–59	10.58	15.05	12.82	1.55*	9.56	12.97	11.27	1.51*
60–64	10.01	13.99	12.04	1.43*	8.71	12.61	10.70	1.49*
65–69	9.57	14.70	12.26	1.57*	8.56	12.93	10.86	1.55*
70–74	10.30	18.82	15.06	1.84*	9.36	17.55	13.94	1.90*
75–79	12.38	19.46	16.65	1.57*	11.40	18.02	15.39	1.58*
80-	11.18	12.83	12.34	1.19	10.26	11.95	11.45	1.20
Total	9.02	10.53	9.78	1.19*	7.97	9.41	8.69	1.21*
*Age-standardized rates*								
WHO 2000–2025	7.66	8.24	7.99	–	6.81	7.38	7.13	–
US 2000	8.44	9.35	8.95	–	7.48	8.37	7.98	–

^a^ Prevalence and incidence are average of those from 2012 to 2015 and reported as per 100,000 persons and per 100,000 person-years, respectively

* *P* < 0.05

**Fig. 1:**
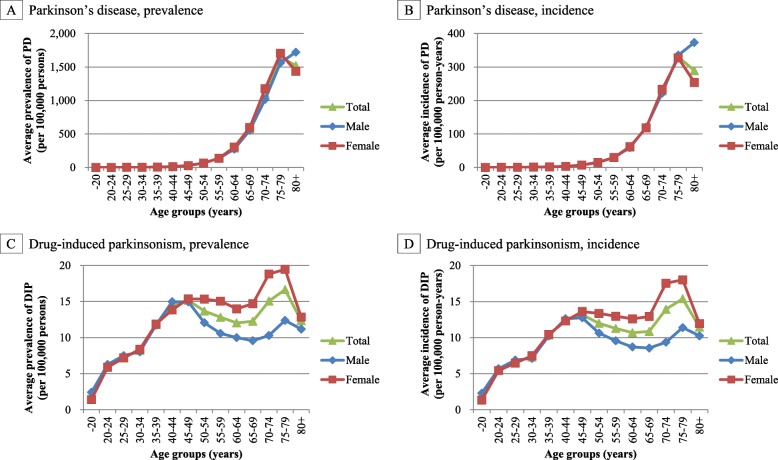
Age- and sex-specific prevalence and incidence of Parkinson’s disease and drug-induced parkinsonism in Korea. *DIP*, drug-induced parkinsonism; *PD*, Parkinson’s disease. The average prevalence per 100,000 persons and average incidence per 100,000 person-years of Parkinson’s disease (**a** and **b**) and drug-induced parkinsonism (**c** and **d**) between 2012 and 2015 in Korea

A higher average prevalence of PD between 2012 and 2015 was observed in the older age groups and the peak prevalence was observed in the over-80 age group, with a higher prevalence in males. In contrast, the average prevalence of DIP between 2012 and 2015 showed a bimodal distribution in the age groups of 45–49 years and 75–79 years, with a higher prevalence in females (Fig. 1).

**Fig. 2:**
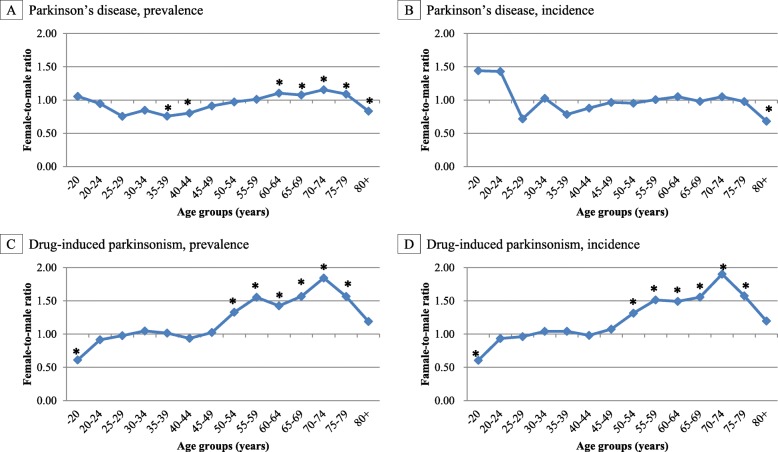
The Female-to-male ratio of prevalence and incidence of Parkinson’s disease and drug-induced parkinsonism in Korea. *DIP*, drug-induced parkinsonism; *PD*, Parkinson’s disease. The average female-to-male ratio of prevalence and incidence of Parkinson’s disease (**a** and **b**) and drug-induced parkinsonism (**c** and **d**) between 2012 and 2015 in Korea (* *P* < 0.05)

The average female-to-male ratio of prevalence of PD between 2012 and 2015 was higher than 1.0 in the age group of 60–79 years, while the average female-to-male ratio of prevalence of DIP between 2012 and 2015 was higher than 1.0 in the age group of 50–79 years and the values of female-to-male ratios were higher than those of PD (Table 1; Fig. 2).

The prevalence of PD significantly increased from 156.90 to 181.33 per 100,000 persons from 2012 to 2015 (*p* < 0.0001; Additional file 1 Table S2). The prevalence of DIP also significantly increased from 7.32 to 15.37 per 100,000 persons from 2012 to 2015 (*p* < 0.0001; Additional file 1 Table S3).

In all of the studied age groups, the trends in the prevalence of PD were quite similar between the sexes by calendar year but an increasing trend of PD prevalence was observed particularly in the age group of over-80 years old. In contrast, the trends in the prevalence of DIP were substantially different among the sexes by calendar year. In both men and women, the prevalence of DIP was the highest in 2015 and the degree of the increase was the highest in middle-aged men and women.

### Incidence of PD and DIP

The mean age of incident patients with PD increased from 70.89 in 2012 to 71.33 in 2015, while the mean age of incident patients with DIP decreased from 48.78 in 2012 to 47.39 in 2015. DIP incident patients were younger than PD incident patients. However, when excluding antipsychotics as a sensitivity analysis, the mean age of incident patients with DIP was 64.31 in 2012 and 63.58 in 2015.

The average incidence of PD between 2012 and 2015 was 34.97 per 100,000 person-years, while the average age-standardized incidence of PD according to the WHO 2000–2025 and US 2000 standard population between 2012 and 2015 was 23.49 and 35.79 per 100,000 person-years, respectively. In contrast, the average incidence of DIP between 2012 and 2015 was 8.69 per 100,000 person-years, and the average age-standardized incidence of DIP according to the WHO 2000–2025 and US 2000 standard population between 2012 and 2015 was 7.13 and 7.98 per 100,000 person-years, respectively.

A higher average incidence of PD between 2012 and 2015 was observed in both male and females in the older age groups. The peak incidence was reached in the age group of over-80 years old, with a higher incidence in males. In contrast, the average incidence of DIP between 2012 and 2015 showed a bimodal distribution in the age group of 45–49 and 75–79 years, with a higher incidence in females (Fig. 1).

The average female-to-male ratio of incidence of PD between 2012 and 2015 was higher than 1.0 in the younger aged groups (younger than 25 years). The older aged groups (over 45 years) had a female-to-male ratio of approximately less than or equal to 1.0. The average female-to-male ratio of the incidence of DIP between 2012 and 2015 was higher than 1.0 in patients aged over 45 years (Table 1; Fig. 2).

The incidence of PD significantly decreased from 35.39 to 33.25 per 100,000 person-years from 2012 to 2015 (*p* < 0.0001; Additional file 1 Table S4). In contrast, the incidence of DIP significantly increased from 7.09 to 13.85 per 100,000 person-years from 2012 to 2015 (*p* < 0.0001; Additional file 1: Table S5).

**Fig. 3:**
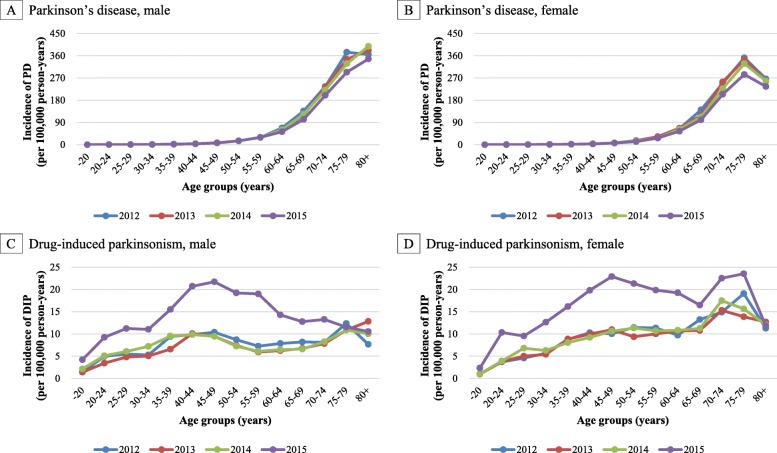
Temporal trends of incidence of Parkinson’s disease and drug-induced parkinsonism in Korea by age group, sex, and calendar year. *DIP*, drug-induced parkinsonism; *PD*, Parkinson’s disease. The incidence per 100,000 person-years of Parkinson’s disease (**a** and **b**) and drug-induced parkinsonism (**c** and **d**) from 2012 to 2015 in Korea

In all age groups, the trends in the incidence of PD were approximately similar between the sexes and calendar year but a decreasing trend of PD incidence was observed particularly in the age group of 75–79 years. In contrast, the trends in the incidence of DIP substantially differed between the sexes and calendar year. In both men and women, the incidence of DIP was highest in 2015 and the degree of this increase was highest in middle-aged men and women (Fig. 3).

### Offending drugs

**Fig. 4:**
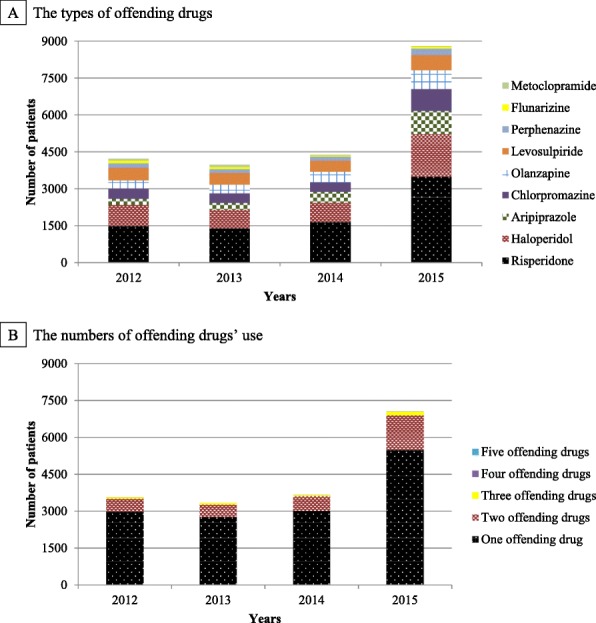
Number of incident patients with drug-induced parkinsonism by the types and numbers of offending drugs in Korea. *DIP*, drug-induced parkinsonism. The types of offending drugs (**a**) and the numbers of offending drugs’ use (**b**) were identified on the day of DIP occurrence from 2012 to 2015 in Korea

In the all of the incident cases, among offending drugs (i.e., levosulpiride, metoclopramide, risperidone, olanzapine, aripiprazole, haloperidol, perphenazine, chlorpromazine, flunarizine), the most commonly used offending drug from 2012 to 2015 was risperidone. In addition, most people used only one offending drug when they were diagnosed as having DIP (Fig. 4).

### Sensitivity analyses

When restricting the study population to patients without any secondary diagnosis of cerebellar ataxia, DIP, or Steele-Richardson-Olszewski syndrome for the sensitivity analysis for PD, the results generally align with the main analysis. However, the prevalence and incidence levels were relatively lower than the main analysis (181.33 per 100,000 persons versus 149.56 per 100,000 persons for the prevalence of all age groups in 2015; 33.25 per 100,000 person-years versus 23.41 per 100,000 person-years for the incidence of all age groups in 2015).

When we excluded antipsychotics as a sensitivity analysis for DIP, the bimodal distributions observed in the prevalence and incidence appeared to disappear. In addition, the trends in the prevalence and incidence of DIP from 2012 to 2015 by age groups and sexes remained rather constant (Additional file 2: Figure S1). The average female-to-male ratios between 2012 and 2015 were larger than those in the primary findings (1.21 versus 1.93 for the female-to-male ratios of incidence of DIP of all age groups). Among all of the incident cases, levosulpiride was the most commonly used drug from 2012 to 2015. Furthermore, most people used only one offending drug when they had a diagnosis of DIP (Additional file 3: Figure S2). The results of the prevalence are not shown due to the similarity in the overall details to those of the incidence.

## Discussion

To the best of our knowledge, this population-based study provides the first direct estimates of the prevalence and incidence of both PD and DIP in Korea using nationwide claims data. Additionally, we observed the trends in the prevalence and incidence of PD and DIP over 4 years, the differences in the prevalence and incidence among age groups and sexes by calendar year, and the types of offending drugs use in incident cases of DIP.

We observed that PD prevalence increased over the period of study, while the PD incidence gradually decreased. In the case of prevalence, incidence, and the trends of PD, results were comparable with those of Taiwan [12]. However, there have been contradictory results regarding the trends in the incidence of PD. Although several studies have reported a stable trend in the incidence of PD, the decreasing trend that was observed in our study. This may be due to lifestyle changes or increased consumption of known PD protective factors, such as coffee, caffeine, statins, or non-steroidal anti-inflammatory drugs in Korea [12–15]. A careful examination is needed in this matter.

In addition, we found that prevalence and incidence of PD were higher in female versus male. There have been inconsistent findings regarding sex difference in prevalence and incidence of PD [16]. Most studies conducted in western countries, including Europe [17, 18], and South/North America [19, 20], reported the male dominance in epidemiological figures [1]. In contrast, some Asian studies, including studies conducted in Korea [5], Japan [21], Taiwan [22], and China [23], reported female dominance or no sex difference in prevalence or incidence of PD [1, 24]. This may indicate that there is a sex difference in the epidemiology of PD between Asian and non-Asian [24, 25]. Further study is needed to explore the reason for a sex difference in the epidemiology of PD between Asian and non-Asian.

Both the prevalence and incidence of DIP showed a tendency to increase rapidly after 2014. This rapid increase after 2014 may be related to the reimbursement change in the diagnosis tool. Positron emission tomography (PET) is an often used tool to diagnosis DIP and two types of PET (I-123 fluorinated N-3-fluoropropyl-2-beta-carboxymethoxy-3-beta-(4-iodophenyl) nortropane (FP-CIT) and F-18 FP-CIT) were included in the benefit list of Korean national health insurance in September 2014. Physicians may have found DIP more than before using the reimbursed diagnosis tool.

We found inconsistent findings in the incidence of DIP when compared to a recent study conducted in United States [3]. The incidence of DIP for all of the age groups in the previous study were lower than those observed in our study, while the incidence of DIP in the older age group were much higher than those in our study [3]. These differences might be related to the differences in the inclusion criteria that were used to identify DIP cases, such as the definition and treatment pattern of the offending drugs, population characteristics (one county study in United States versus population-based Asian study), and time (1976–2005 versus 2011–2015).

Our results indicate that the prevalence and incidence of DIP were higher in females and in people of old age. Our finding of female dominance in DIP is consistent with a previous finding [3]. This may be related to the drug utilization pattern in females and people of old age [3]. In addition, a possible explanation of the observed female dominance in patients with DIP is the suppression of dopamine receptors by estrogen [7]. However, we also found that the DIP incidence increased sharply after 2014, especially in the middle-aged group. Further studies regarding the factors that affect the prevalence and incidence of DIP are needed.

The most common class of drugs related with DIP in this study was antipsychotics. This finding is in line with the findings of the previous study [3]. However, unlike the previous study [3], we found that there was a higher involvement of atypical antipsychotics with DIP than typical antipsychotics. The dose or intensity of atypical antipsychotics might play a role in this higher involvement [26]. However, further research is needed because we did not consider the dose or intensity of antipsychotics in this analysis.

The bimodal distribution in the prevalence and incidence of DIP seemed to be related to DIP that occurred with the use of antipsychotics in this study. Because when we excluded antipsychotics from the analysis, the bimodal distribution disappeared. We also observed the bimodal distribution peaked in midlife and elderly. One of the factors that may contribute to this trend in the distribution may be related with the higher use of antipsychotics in midlife and elderly, which has been reported in previous study [27].

In order to examine the relationship between DIP and propulsives, we excluded antipsychotics as a sensitivity analysis. When antipsychotics were excluded from the analysis, the female-to-male ratio increased and levosulpiride was found to be the drug that was most frequently related with DIP. A previous study from Korea also emphasized the frequent prescriptions of levosulpiride and the importance of levosulpiride-induced parkinsonism [28]. In contrast, the frequency of metoclopramide in DIP incident cases decreased after 2013. This may be because the Ministry of Food and Drug Safety distributed a safety letter regarding metoclopramide-induced extrapyramidal syndrome in 2013.

The standard populations used for estimating age-standardized prevalence and incidence were WHO 2000–2025 and US 2000. In the WHO 2000–2025 population, 8.2% were older age group (≥65 years) but percentage of female was not available. In the US 2000 population, 12.6% were older age group (≥65 years) and 51.1% were female. In the Korean population, 11.4% in 2012 and 12.9% in 2015 were older age group (≥65 years) which was similar to the US 2000 population and slightly different from the WHO 2000–2025 population. Percentages of female in both 2012 and 2015 were 50.0%, which was also similar to the US 2000 population. We used the two most commonly utilized standard populations in estimating age-standardized prevalence and incidence to increase comparability of results across different studies. The differences in share of older age group (≥65 years) between the WHO 2000–2025 population and the US 2000 population seemed to contribute to the differences in age-standardized prevalence and incidence using these populations. Although the share of older age group of the WHO 2000–2025 was somewhat lower than the US 2000, it is meaningful to use this population in terms of increasing comparability across studies from different countries because the WHO 2000–2025 population is based on the average age structure of the world populations over the period of 2000–2025 [10].

This study has several strengths worth mentioning. First, this population-based study provides the first direct estimates of the prevalence and incidence of both PD and DIP in Korea using nationwide claims data. Second, we identified patients with PD and DIP using operational definitions reflecting the expert opinions of neurologists in Korea as well as the published literature [2, 5, 7]. Because claims we used were electronically submitted by healthcare providers for reimbursement purposes, opinions of medical experts were important to identify patients with PD and DIP based on current clinical practice. Third, sensitivity analyses were performed to assess whether excluding patients with PD that were suspected to have additional neurological disorders (i.e., cerebellar ataxia, DIP, and Steele-Richardson-Olszewski syndrome) and whether excluding cases of DIP linked to antipsychotics affected the overall epidemiological trends.

Our study has several limitations. First, we could not determine whether the diagnosis of PD or DIP was confirmed through imaging, because the test results are not available in claims database. Second, the prevalence and incidence of PD and DIP in our study may be underestimated, as some patients may not seek medical care. Notably, patients prescribed with antiemetics have a low awareness of the risk of antiemetics-induced parkinsonism, resulting in low rates of reporting of their movement symptoms. Third, since we used claims data that were collected for reimbursement purposes, the validity of the diagnose codes used to identify PD and DIP may be questioned and they have not been established. However, we used operational definitions based on the expert opinions of neurologists and on previous studies [2, 5, 7]. Lastly, we did not examine the statistical association between DIP and offending drugs. We only examined the concomitant use of offending drugs and DIP occurrence. Therefore, the relationship between DIP and offending drugs should be interpreted in caution.

## Conclusions

Our study suggests that the incidence of PD has gradually decreased whereas the prevalence of PD increased from 2012 to 2015. In addition, both the prevalence and incidence of DIP increased from 2012 to 2015, particularly after 2014. The most commonly used offending drug was risperidone. Longer-term trends need to be evaluated and further studies are warranted to explore the possible causes of the observed epidemiological trends.

## Supplementary information

**Additional file 1: Table S1.** Medications specifically used for Parkinson’s disease. **Table S2.** Frequencies and prevalence of Parkinson’s disease in Korea from 2012 to 2015. **Table S3.** Frequencies and prevalence of drug-induced parkinsonism in Korea from 2012 to 2015. **Table S4.** Frequencies and incidence of Parkinson’s disease in Korea from 2012 to 2015. **Table S5.** Frequencies and incidence of drug-induced parkinsonism in Korea from 2012 to 2015.

**Additional file 2: Figure S1.** Sensitivity analysis. Incidence of drug-induced parkinsonism in Korea.

**Additional file 3: Figure S2.** Sensitivity analysis. Number of incident patients with drug-induced parkinsonism by the types and numbers of offending drugs in Korea

## Data Availability

The data that support the findings of this study are available from Health Insurance Review and Assessment but restrictions apply to the availability of these data, which were used under license for the current study, and so are not publicly available.

## Abbreviations

DIP: Drug-induced parkinsonism
HIRA: Health Insurance Review and Assessment
ICD-10: *International Classification of Diseases 10th Revision*
KCD: Korean Standard Classification of Diseases
PD: Parkinson’s disease
