# Supplementary material for: Prevalence and incidence of Parkinson’s disease and drug-induced parkinsonism in Korea
Source: BMC Public Health. 2019 Oct 22;19:1328. doi: 10.1186/s12889-019-7664-6 (PMC6805681; doi:10.1186/s12889-019-7664-6)
Supplement: Supplementary file 1 — Additional file 1: Table S1. Medications specifically used for Parkinson’s disease. Table S2. Frequencies and prevalence of Parkinson’s disease in Korea from 2012 to 2015. Table S3. Frequencies and prevalence of drug-induced parkinsonism in Korea from 2012 to 2015. Table S4. Frequencies and incidence of Parkinson’s disease in Korea from 2012 to 2015. Table S5. Frequencies and incidence of drug-induced parkinsonism in Korea from 2012 to 2015. [file 12889_2019_7664_MOESM1_ESM.docx]

**Supplementary Online Content**

eFigure 1. Sensitivity analysis. Incidence of drug-induced parkinsonism in Korea

eFigure 2. Sensitivity analysis. Number of incident patients with drug-induced parkinsonism by the types and numbers of offending drugs in Korea

eTable 1. Medications specifically used for Parkinson’s disease

eTable 2. Frequencies and prevalence of Parkinson’s disease in Korea from 2012 to 2015

eTable 3. Frequencies and prevalence of drug-induced parkinsonism in Korea from 2012 to 2015

eTable 4. Frequencies and incidence of Parkinson’s disease in Korea from 2012 to 2015

eTable 5. Frequencies and incidence of drug-induced parkinsonism in Korea from 2012 to 2015

This supplementary material has been provided by the authors to give readers additional information about their work.

**eTable 1. Medications specifically used for Parkinson’s disease**

| **ATC code** | **Name** |
| --- | --- |
| N04BX02 | Entacapone |
| N04BA02 | Levodopa and decarboxylase inhibitor |
| N04BA03 | Levodopa, decarboxylase inhibitor and COMT inhibitor |
| N04BC02 | Pergolide |
| N04BC05 | Pramipexole |
| N04BD02 | Rasagiline |
| N04BC04 | Ropinirole |
| N04BD01 | Selegiline |
| COMT, Catechol-O-methyltransferase. | |

**eTable 2. Frequencies and prevalence of Parkinson’s disease in Korea from 2012 to 2015**

| **Calendar year** | **Age groups**  **(years)** | **Total** | | **Male** | | **Female** | | **Female-to-male ratio**  **(*P* value)** |
| --- | --- | --- | --- | --- | --- | --- | --- | --- |
|  |  | No. | Prevalence  (95% CI) | No. | Prevalence  (95% CI) | No. | Prevalence  (95% CI) |  |
| 2012 | 0-19 | 18 | 0.16 (0.16-0.16) | 9 | 0.15 (0.15-0.15) | 9 | 0.17 (0.17-0.17) | 1.09 (0.850) |
|  | 20-24 | 27 | 0.83 (0.83-0.83) | 17 | 0.99 (0.99-0.99) | 10 | 0.65 (0.65-0.65) | 0.66 (0.285) |
|  | 25-29 | 52 | 1.53 (1.53-1.54) | 27 | 1.54 (1.54-1.55) | 25 | 1.52 (1.52-1.52) | 0.99 (0.958) |
|  | 30-34 | 123 | 3.07 (3.07-3.07) | 64 | 3.13 (3.12-3.13) | 59 | 3.01 (3.00-3.01) | 0.96 (0.830) |
|  | 35-39 | 263 | 6.38 (6.38-6.38) | 154 | 7.35 (7.35-7.35) | 109 | 5.38 (5.37-5.38) | 0.73 (0.012) |
|  | 40-44 | 538 | 11.78 (11.78-11.79) | 304 | 13.12 (13.12-13.13) | 234 | 10.41 (10.40-10.41) | 0.79 (0.008) |
|  | 45-49 | 1190 | 28.69 (28.69-28.70) | 630 | 29.70 (29.7-29.71) | 560 | 27.63 (27.63-27.64) | 0.93 (0.213) |
|  | 50-54 | 2873 | 66.98 (66.97-66.99) | 1470 | 68.04 (68.03-68.05) | 1403 | 65.91 (65.90-65.92) | 0.97 (0.394) |
|  | 55-59 | 4482 | 137.99 (137.98-138.01) | 2155 | 133.35 (133.33-133.36) | 2327 | 142.59 (142.58-142.61) | 1.07 (0.025) |
|  | 60-64 | 6969 | 297.74 (297.72-297.76) | 3258 | 284.32 (284.29-284.35) | 3711 | 310.62 (310.59-310.65) | 1.09 (<0.001) |
|  | 65-69 | 11467 | 610.88 (610.85-610.92) | 5020 | 570.70 (570.65-570.75) | 6447 | 646.32 (646.27-646.37) | 1.13 (<0.001) |
|  | 70-74 | 18326 | 1082.94 (1082.89-1082.99) | 7312 | 990.85 (990.78-990.92) | 11014 | 1154.16 (1154.09-1154.23) | 1.16 (<0.001) |
|  | 75-79 | 18503 | 1604.43 (1604.35-1604.50) | 6758 | 1517.57 (1517.46-1517.69) | 11745 | 1659.06 (1658.97-1659.16) | 1.09 (<0.001) |
|  | 80- | 14161 | 1365.32 (1365.25-1365.39) | 4721 | 1571.06 (1570.91-1571.20) | 9440 | 1281.40 (1281.32-1281.49) | 0.82 (<0.001) |
|  | Total | 78992 | 156.90 (156.90-156.90) | 31899 | 126.65 (126.64-126.65) | 47093 | 187.19 (187.18-187.20) | 1.48 (<0.001) |
| 2013 | 0-19 | 22 | 0.20 (0.20-0.20) | 13 | 0.23 (0.23-0.23) | 9 | 0.17 (0.17-0.17) | 0.75 (0.513) |
|  | 20-24 | 43 | 1.28 (1.28-1.29) | 22 | 1.24 (1.24-1.24) | 21 | 1.33 (1.33-1.33) | 1.07 (0.822) |
|  | 25-29 | 53 | 1.64 (1.64-1.64) | 30 | 1.80 (1.80-1.80) | 23 | 1.48 (1.47-1.48) | 0.82 (0.474) |
|  | 30-34 | 140 | 3.46 (3.46-3.47) | 77 | 3.73 (3.73-3.73) | 63 | 3.18 (3.18-3.19) | 0.85 (0.351) |
|  | 35-39 | 240 | 6.04 (6.03-6.04) | 141 | 6.97 (6.97-6.98) | 99 | 5.06 (5.06-5.07) | 0.73 (0.014) |
|  | 40-44 | 541 | 11.86 (11.86-11.87) | 291 | 12.54 (12.54-12.55) | 250 | 11.16 (11.15-11.16) | 0.89 (0.175) |
|  | 45-49 | 1203 | 28.67 (28.67-28.68) | 637 | 29.87 (29.86-29.88) | 566 | 27.43 (27.42-27.44) | 0.92 (0.140) |
|  | 50-54 | 2907 | 67.04 (67.03-67.04) | 1473 | 67.33 (67.32-67.34) | 1434 | 66.74 (66.73-66.75) | 0.99 (0.812) |
|  | 55-59 | 4741 | 137.43 (137.41-137.44) | 2313 | 134.36 (134.34-134.38) | 2428 | 140.48 (140.46-140.50) | 1.05 (0.125) |
|  | 60-64 | 7139 | 294.62 (294.59-294.64) | 3318 | 279.57 (279.54-279.60) | 3821 | 309.06 (309.03-309.09) | 1.11 (<0.001) |
|  | 65-69 | 11071 | 576.54 (576.51-576.58) | 4991 | 549.66 (549.61-549.71) | 6080 | 600.66 (600.61-600.71) | 1.09 (<0.001) |
|  | 70-74 | 19982 | 1127.45 (1127.40-1127.50) | 8011 | 1028.32 (1028.25-1028.40) | 11971 | 1205.20 (1205.13-1205.27) | 1.17 (<0.001) |
|  | 75-79 | 20266 | 1658.10 (1658.03-1658.18) | 7575 | 1579.52 (1579.41-1579.64) | 12691 | 1708.85 (1708.75-1708.94) | 1.08 (<0.001) |
|  | 80- | 16718 | 1498.34 (1498.27-1498.41) | 5563 | 1699.23 (1699.08-1699.36) | 11155 | 1414.92 (1414.84-1415) | 0.83 (<0.001) |
|  | Total | 85066 | 168.25 (168.25-168.25) | 34455 | 136.28 (136.27-136.28) | 50611 | 200.23 (200.23-200.24) | 1.47 (<0.001) |
| 2014 | 0-19 | 26 | 0.24 (0.24-0.24) | 13 | 0.23 (0.23-0.23) | 13 | 0.25 (0.25-0.25) | 1.08 (0.839) |
|  | 20-24 | 40 | 1.16 (1.16-1.17) | 22 | 1.21 (1.21-1.21) | 18 | 1.12 (1.11-1.12) | 0.92 (0.802) |
|  | 25-29 | 50 | 1.59 (1.59-1.59) | 32 | 1.96 (1.96-1.96) | 18 | 1.19 (1.18-1.19) | 0.61 (0.086) |
|  | 30-34 | 135 | 3.40 (3.40-3.40) | 75 | 3.70 (3.69-3.70) | 60 | 3.09 (3.08-3.09) | 0.84 (0.297) |
|  | 35-39 | 250 | 6.45 (6.45-6.45) | 145 | 7.35 (7.35-7.36) | 105 | 5.52 (5.51-5.52) | 0.75 (0.025) |
|  | 40-44 | 542 | 12.02 (12.02-12.02) | 304 | 13.24 (13.24-13.25) | 238 | 10.75 (10.75-10.75) | 0.81 (0.016) |
|  | 45-49 | 1270 | 29.52 (29.52-29.53) | 672 | 30.84 (30.83-30.85) | 598 | 28.17 (28.17-28.18) | 0.91 (0.108) |
|  | 50-54 | 2893 | 66.93 (66.93-66.94) | 1477 | 67.61 (67.60-67.62) | 1416 | 66.25 (66.23-66.26) | 0.98 (0.584) |
|  | 55-59 | 4992 | 135.71 (135.70-135.72) | 2521 | 137.29 (137.27-137.31) | 2471 | 134.14 (134.12-134.15) | 0.98 (0.411) |
|  | 60-64 | 7332 | 290.82 (290.80-290.84) | 3376 | 273.09 (273.06-273.12) | 3956 | 307.87 (307.84-307.90) | 1.13 (<0.001) |
|  | 65-69 | 11452 | 570.10 (570.06-570.13) | 5383 | 561.87 (561.83-561.92) | 6069 | 577.59 (577.55-577.64) | 1.03 (0.139) |
|  | 70-74 | 20124 | 1127.34 (1127.29-1127.39) | 8174 | 1035.47 (1035.40-1035.54) | 11950 | 1200.17 (1200.10-1200.24) | 1.16 (<0.001) |
|  | 75-79 | 21719 | 1675.42 (1675.35-1675.49) | 8220 | 1592.92 (1592.81-1593.02) | 13499 | 1729.98 (1729.89-1730.07) | 1.09 (<0.001) |
|  | 80- | 19309 | 1600.11 (1600.04-1600.18) | 6513 | 1810.21 (1810.06-1810.34) | 12796 | 1510.86 (1510.78-1510.94) | 0.83 (<0.001) |
|  | Total | 90134 | 177.56 (177.55-177.56) | 36927 | 145.53 (145.52-145.53) | 53207 | 209.57 (209.56-209.58) | 1.44 (<0.001) |
| 2015 | 0-19 | 22 | 0.21 (0.21-0.21) | 10 | 0.18 (0.18-0.19) | 12 | 0.24 (0.24-0.24) | 1.29 (0.547) |
|  | 20-24 | 42 | 1.20 (1.20-1.20) | 21 | 1.13 (1.13-1.13) | 21 | 1.27 (1.27-1.28) | 1.13 (0.690) |
|  | 25-29 | 50 | 1.60 (1.60-1.60) | 32 | 1.96 (1.96-1.96) | 18 | 1.20 (1.20-1.20) | 0.61 (0.092) |
|  | 30-34 | 126 | 3.32 (3.32-3.32) | 74 | 3.81 (3.81-3.81) | 52 | 2.81 (2.80-2.81) | 0.74 (0.090) |
|  | 35-39 | 260 | 6.70 (6.70-6.70) | 145 | 7.34 (7.34-7.35) | 115 | 6.03 (6.03-6.03) | 0.82 (0.114) |
|  | 40-44 | 549 | 12.45 (12.44-12.45) | 324 | 14.43 (14.43-14.44) | 225 | 10.39 (10.39-10.39) | 0.72 (<0.001) |
|  | 45-49 | 1281 | 29.29 (29.29-29.30) | 688 | 31.07 (31.06-31.08) | 593 | 27.47 (27.47-27.48) | 0.88 (0.028) |
|  | 50-54 | 2728 | 63.94 (63.94-63.95) | 1419 | 65.70 (65.69-65.71) | 1309 | 62.15 (62.13-62.16) | 0.95 (0.147) |
|  | 55-59 | 5301 | 136.53 (136.52-136.55) | 2710 | 139.88 (139.86-139.90) | 2591 | 133.20 (133.18-133.22) | 0.95 (0.075) |
|  | 60-64 | 7472 | 275.20 (275.18-275.22) | 3512 | 263.73 (263.70-263.75) | 3960 | 286.24 (286.21-286.27) | 1.09 (<0.001) |
|  | 65-69 | 11562 | 548.10 (548.07-548.13) | 5398 | 533.99 (533.94-534.03) | 6164 | 561.08 (561.04-561.13) | 1.05 (0.008) |
|  | 70-74 | 19333 | 1087.74 (1087.70-1087.79) | 8053 | 1016.81 (1016.74-1016.88) | 11280 | 1144.76 (1144.69-1144.82) | 1.13 (<0.001) |
|  | 75-79 | 22480 | 1660.73 (1660.66-1660.80) | 8581 | 1572.09 (1571.98-1572.19) | 13899 | 1720.63 (1720.54-1720.72) | 1.09 (<0.001) |
|  | 80- | 21187 | 1614.76 (1614.70-1614.83) | 7190 | 1804.50 (1804.37-1804.63) | 13997 | 1532.02 (1531.94-1532.10) | 0.85 (<0.001) |
|  | Total | 92393 | 181.33 (181.33-181.34) | 38157 | 149.88 (149.88-149.89) | 54236 | 212.74 (212.74-212.75) | 1.42 (<0.001) |
| * The prevalence is reported as per 100,000 persons for patients with Parkinson’s disease. | | | | | | | | |

eTable 3. Frequencies and prevalence of drug-induced parkinsonism in Korea from 2012 to 2015

| **Calendar year** | **Age groups**  **(years)** | **Total** | | **Male** | | **Female** | | **Female-to-male ratio**  **(*P* value)** |
| --- | --- | --- | --- | --- | --- | --- | --- | --- |
|  |  | No. | Prevalence  (95% CI) | No. | Prevalence  (95% CI) | No. | Prevalence  (95% CI) |  |
| 2012 | 0-19 | 139 | 1.24 (1.24-1.24) | 85 | 1.45 (1.45-1.45) | 54 | 1.01 (1.01-1.01) | 0.69 (0.035) |
|  | 20-24 | 145 | 4.46 (4.46-4.46) | 88 | 5.13 (5.13-5.14) | 57 | 3.70 (3.70-3.71) | 0.72 (0.054) |
|  | 25-29 | 174 | 5.13 (5.13-5.13) | 96 | 5.49 (5.49-5.50) | 78 | 4.75 (4.75-4.75) | 0.86 (0.341) |
|  | 30-34 | 225 | 5.61 (5.61-5.61) | 110 | 5.37 (5.37-5.37) | 115 | 5.86 (5.86-5.86) | 1.09 (0.513) |
|  | 35-39 | 382 | 9.27 (9.26-9.27) | 198 | 9.45 (9.45-9.45) | 184 | 9.07 (9.07-9.08) | 0.96 (0.692) |
|  | 40-44 | 470 | 10.29 (10.29-10.30) | 238 | 10.27 (10.27-10.28) | 232 | 10.32 (10.31-10.32) | 1.00 (0.963) |
|  | 45-49 | 433 | 10.44 (10.44-10.44) | 227 | 10.70 (10.70-10.71) | 206 | 10.16 (10.16-10.17) | 0.95 (0.592) |
|  | 50-54 | 446 | 10.40 (10.39-10.40) | 193 | 8.93 (8.93-8.94) | 253 | 11.88 (11.88-11.89) | 1.33 (0.003) |
|  | 55-59 | 313 | 9.64 (9.63-9.64) | 120 | 7.43 (7.42-7.43) | 193 | 11.83 (11.82-11.83) | 1.59 (<0.001) |
|  | 60-64 | 212 | 9.06 (9.05-9.06) | 94 | 8.20 (8.20-8.21) | 118 | 9.88 (9.87-9.88) | 1.20 (0.179) |
|  | 65-69 | 220 | 11.72 (11.72-11.73) | 76 | 8.64 (8.63-8.65) | 144 | 14.44 (14.43-14.44) | 1.67 (<0.001) |
|  | 70-74 | 216 | 12.76 (12.76-12.77) | 67 | 9.08 (9.07-9.09) | 149 | 15.61 (15.61-15.62) | 1.72 (<0.001) |
|  | 75-79 | 199 | 17.26 (17.25-17.26) | 56 | 12.58 (12.56-12.59) | 143 | 20.20 (20.19-20.21) | 1.61 (0.002) |
|  | 80- | 112 | 10.80 (10.79-10.80) | 24 | 7.99 (7.98-8.00) | 88 | 11.95 (11.94-11.95) | 1.50 (0.078) |
|  | Total | 3686 | 7.32 (7.32-7.32) | 1672 | 6.64 (6.64-6.64) | 2014 | 8.01 (8.00-8.01) | 1.21 (<0.001) |
| 2013 | 0-19 | 145 | 1.32 (1.32-1.32) | 88 | 1.54 (1.54-1.54) | 57 | 1.09 (1.08-1.09) | 0.70 (0.039) |
|  | 20-24 | 140 | 4.18 (4.18-4.18) | 71 | 4.01 (4.01-4.01) | 69 | 4.37 (4.37-4.37) | 1.09 (0.609) |
|  | 25-29 | 174 | 5.39 (5.39-5.39) | 85 | 5.10 (5.09-5.1) | 89 | 5.71 (5.71-5.71) | 1.12 (0.454) |
|  | 30-34 | 244 | 6.04 (6.03-6.04) | 118 | 5.72 (5.71-5.72) | 126 | 6.37 (6.37-6.37) | 1.11 (0.398) |
|  | 35-39 | 383 | 9.63 (9.63-9.63) | 178 | 8.80 (8.80-8.81) | 205 | 10.48 (10.48-10.49) | 1.19 (0.088) |
|  | 40-44 | 554 | 12.15 (12.14-12.15) | 289 | 12.46 (12.45-12.46) | 265 | 11.83 (11.82-11.83) | 0.95 (0.543) |
|  | 45-49 | 515 | 12.27 (12.27-12.28) | 255 | 11.96 (11.95-11.96) | 260 | 12.60 (12.60-12.61) | 1.05 (0.552) |
|  | 50-54 | 448 | 10.33 (10.33-10.33) | 197 | 9.00 (9.00-9.01) | 251 | 11.68 (11.68-11.69) | 1.30 (0.006) |
|  | 55-59 | 353 | 10.23 (10.23-10.24) | 129 | 7.49 (7.49-7.50) | 224 | 12.96 (12.95-12.97) | 1.73 (<0.001) |
|  | 60-64 | 240 | 9.90 (9.90-9.91) | 92 | 7.75 (7.75-7.76) | 148 | 11.97 (11.96-11.98) | 1.54 (0.001) |
|  | 65-69 | 202 | 10.52 (10.52-10.52) | 73 | 8.04 (8.03-8.05) | 129 | 12.74 (12.74-12.75) | 1.59 (0.002) |
|  | 70-74 | 232 | 13.09 (13.08-13.10) | 67 | 8.60 (8.59-8.61) | 165 | 16.61 (16.60-16.62) | 1.93 (<0.001) |
|  | 75-79 | 173 | 14.15 (14.15-14.16) | 59 | 12.30 (12.29-12.31) | 114 | 15.35 (15.34-15.36) | 1.25 (0.167) |
|  | 80- | 155 | 13.89 (13.88-13.90) | 47 | 14.36 (14.34-14.37) | 108 | 13.70 (13.69-13.71) | 0.95 (0.789) |
|  | Total | 3958 | 7.83 (7.83-7.83) | 1748 | 6.91 (6.91-6.91) | 2210 | 8.74 (8.74-8.74) | 1.26 (<0.001) |
| 2014 | 0-19 | 183 | 1.71 (1.71-1.71) | 126 | 2.26 (2.26-2.27) | 57 | 1.11 (1.11-1.11) | 0.49 (<0.001) |
|  | 20-24 | 179 | 5.21 (5.21-5.22) | 109 | 5.99 (5.98-5.99) | 70 | 4.34 (4.34-4.34) | 0.72 (0.035) |
|  | 25-29 | 230 | 7.30 (7.30-7.30) | 117 | 7.16 (7.15-7.16) | 113 | 7.45 (7.44-7.45) | 1.04 (0.764) |
|  | 30-34 | 317 | 7.98 (7.98-7.98) | 173 | 8.53 (8.52-8.53) | 144 | 7.41 (7.41-7.41) | 0.87 (0.212) |
|  | 35-39 | 409 | 10.55 (10.55-10.56) | 223 | 11.31 (11.30-11.31) | 186 | 9.77 (9.77-9.78) | 0.86 (0.142) |
|  | 40-44 | 543 | 12.04 (12.04-12.04) | 291 | 12.68 (12.67-12.68) | 252 | 11.38 (11.38-11.39) | 0.90 (0.210) |
|  | 45-49 | 554 | 12.88 (12.88-12.88) | 273 | 12.53 (12.52-12.53) | 281 | 13.24 (13.23-13.24) | 1.06 (0.516) |
|  | 50-54 | 486 | 11.24 (11.24-11.25) | 192 | 8.79 (8.78-8.79) | 294 | 13.75 (13.75-13.76) | 1.57 (<0.001) |
|  | 55-59 | 382 | 10.38 (10.38-10.39) | 137 | 7.46 (7.46-7.46) | 245 | 13.30 (13.29-13.30) | 1.78 (<0.001) |
|  | 60-64 | 266 | 10.55 (10.55-10.55) | 98 | 7.93 (7.92-7.93) | 168 | 13.07 (13.07-13.08) | 1.65 (<0.001) |
|  | 65-69 | 214 | 10.65 (10.65-10.66) | 76 | 7.93 (7.93-7.94) | 138 | 13.13 (13.13-13.14) | 1.66 (<0.001) |
|  | 70-74 | 258 | 14.45 (14.45-14.46) | 73 | 9.25 (9.24-9.25) | 185 | 18.58 (18.57-18.59) | 2.01 (<0.001) |
|  | 75-79 | 192 | 14.81 (14.80-14.82) | 61 | 11.82 (11.81-11.83) | 131 | 16.79 (16.78-16.80) | 1.42 (0.023) |
|  | 80- | 148 | 12.26 (12.26-12.27) | 38 | 10.56 (10.55-10.57) | 110 | 12.99 (12.98-13.00) | 1.23 (0.271) |
|  | Total | 4361 | 8.59 (8.59-8.59) | 1987 | 7.83 (7.83-7.83) | 2374 | 9.35 (9.35-9.35) | 1.19 (<0.001) |
| 2015 | 0-19 | 362 | 3.47 (3.47-3.47) | 239 | 4.42 (4.41-4.42) | 123 | 2.45 (2.45-2.45) | 0.55 (<0.001) |
|  | 20-24 | 370 | 10.54 (10.54-10.55) | 186 | 9.99 (9.98-9.99) | 184 | 11.17 (11.17-11.18) | 1.12 (0.281) |
|  | 25-29 | 362 | 11.56 (11.56-11.57) | 200 | 12.27 (12.26-12.27) | 162 | 10.80 (10.80-10.81) | 0.88 (0.228) |
|  | 30-34 | 501 | 13.20 (13.19-13.20) | 243 | 12.51 (12.50-12.51) | 258 | 13.92 (13.91-13.92) | 1.11 (0.231) |
|  | 35-39 | 689 | 17.75 (17.75-17.76) | 343 | 17.37 (17.37-17.38) | 346 | 18.14 (18.14-18.15) | 1.04 (0.569) |
|  | 40-44 | 1023 | 23.20 (23.19-23.20) | 550 | 24.50 (24.49-24.51) | 473 | 21.84 (21.84-21.85) | 0.89 (0.067) |
|  | 45-49 | 1096 | 25.06 (25.06-25.07) | 546 | 24.66 (24.65-24.66) | 550 | 25.48 (25.47-25.49) | 1.03 (0.585) |
|  | 50-54 | 970 | 22.74 (22.73-22.74) | 464 | 21.48 (21.48-21.49) | 506 | 24.02 (24.02-24.03) | 1.12 (0.082) |
|  | 55-59 | 816 | 21.02 (21.01-21.02) | 386 | 19.92 (19.92-19.93) | 430 | 22.11 (22.10-22.11) | 1.11 (0.138) |
|  | 60-64 | 506 | 18.64 (18.63-18.64) | 215 | 16.14 (16.14-16.15) | 291 | 21.03 (21.03-21.04) | 1.30 (0.003) |
|  | 65-69 | 341 | 16.17 (16.16-16.17) | 138 | 13.65 (13.64-13.66) | 203 | 18.48 (18.47-18.49) | 1.35 (0.006) |
|  | 70-74 | 354 | 19.92 (19.91-19.92) | 113 | 14.27 (14.26-14.28) | 241 | 24.46 (24.45-24.47) | 1.71 (<0.001) |
|  | 75-79 | 276 | 20.39 (20.38-20.40) | 70 | 12.82 (12.81-12.83) | 206 | 25.50 (25.49-25.51) | 1.99 (<0.001) |
|  | 80- | 163 | 12.42 (12.42-12.43) | 47 | 11.80 (11.79-11.81) | 116 | 12.70 (12.69-12.70) | 1.08 (0.670) |
|  | Total | 7829 | 15.37 (15.36-15.37) | 3740 | 14.69 (14.69-14.69) | 4089 | 16.04 (16.04-16.04) | 1.09 (<0.001) |
| * The prevalence is reported as per 100,000 persons for patients with drug-induced parkinsonism. | | | | | | | | |

eTable 4. Frequencies and incidence of Parkinson’s disease in Korea from 2012 to 2015

| **Calendar year** | **Age groups**  **(years)** | **Total** | | **Male** | | **Female** | | **Female-to-male ratio**  **(*P* value)** |
| --- | --- | --- | --- | --- | --- | --- | --- | --- |
|  |  | No. | Incidence  (95% CI) | No. | Incidence  (95% CI) | No. | Incidence  (95% CI) |  |
| 2012 | 0-19 | 11 | 0.10 (0.10-0.10) | 6 | 0.10 (0.10-0.10) | 5 | 0.09 (0.09-0.09) | 0.91 (0.877) |
|  | 20-24 | 7 | 0.22 (0.22-0.22) | 5 | 0.29 (0.29-0.29) | 2 | 0.13 (0.13-0.13) | 0.45 (0.321) |
|  | 25-29 | 15 | 0.44 (0.44-0.44) | 7 | 0.40 (0.40-0.40) | 8 | 0.49 (0.49-0.49) | 1.22 (0.704) |
|  | 30-34 | 45 | 1.12 (1.12-1.12) | 18 | 0.88 (0.88-0.88) | 27 | 1.38 (1.37-1.38) | 1.57 (0.138) |
|  | 35-39 | 86 | 2.09 (2.08-2.09) | 52 | 2.48 (2.48-2.48) | 34 | 1.68 (1.68-1.68) | 0.68 (0.074) |
|  | 40-44 | 152 | 3.33 (3.33-3.33) | 87 | 3.75 (3.75-3.76) | 65 | 2.89 (2.89-2.89) | 0.77 (0.109) |
|  | 45-49 | 302 | 7.28 (7.28-7.28) | 152 | 7.17 (7.16-7.17) | 150 | 7.40 (7.40-7.41) | 1.03 (0.779) |
|  | 50-54 | 705 | 16.44 (16.43-16.44) | 343 | 15.88 (15.87-15.88) | 362 | 17.01 (17.00-17.01) | 1.07 (0.362) |
|  | 55-59 | 1004 | 30.91 (30.91-30.92) | 473 | 29.27 (29.26-29.28) | 531 | 32.54 (32.53-32.55) | 1.11 (0.094) |
|  | 60-64 | 1553 | 66.35 (66.34-66.36) | 775 | 67.63 (67.62-67.65) | 778 | 65.12 (65.11-65.13) | 0.96 (0.456) |
|  | 65-69 | 2606 | 138.83 (138.81-138.85) | 1198 | 136.19 (136.17-136.22) | 1408 | 141.15 (141.13-141.18) | 1.04 (0.362) |
|  | 70-74 | 4115 | 243.17 (243.15-243.19) | 1737 | 235.38 (235.35-235.42) | 2378 | 249.19 (249.16-249.22) | 1.06 (0.070) |
|  | 75-79 | 4156 | 360.37 (360.34-360.41) | 1666 | 374.12 (374.06-374.17) | 2490 | 351.73 (351.69-351.77) | 0.94 (0.051) |
|  | 80- | 3060 | 295.03 (295.00-295.06) | 1097 | 365.06 (364.99-365.13) | 1963 | 266.46 (266.42-266.50) | 0.73 (<0.001) |
|  | Total | 17817 | 35.39 (35.39-35.39) | 7616 | 30.24 (30.24-30.24) | 10201 | 40.55 (40.55-40.55) | 1.34 (<0.001) |
| 2013 | 0-19 | 11 | 0.10 (0.10-0.10) | 7 | 0.12 (0.12-0.12) | 4 | 0.08 (0.08-0.08) | 0.62 (0.444) |
|  | 20-24 | 23 | 0.69 (0.69-0.69) | 9 | 0.51 (0.51-0.51) | 14 | 0.89 (0.89-0.89) | 1.75 (0.187) |
|  | 25-29 | 19 | 0.59 (0.59-0.59) | 12 | 0.72 (0.72-0.72) | 7 | 0.45 (0.45-0.45) | 0.62 (0.317) |
|  | 30-34 | 49 | 1.21 (1.21-1.21) | 25 | 1.21 (1.21-1.21) | 24 | 1.21 (1.21-1.22) | 1.00 (0.995) |
|  | 35-39 | 62 | 1.56 (1.56-1.56) | 36 | 1.78 (1.78-1.78) | 26 | 1.33 (1.33-1.33) | 0.75 (0.255) |
|  | 40-44 | 160 | 3.51 (3.51-3.51) | 75 | 3.23 (3.23-3.24) | 85 | 3.79 (3.79-3.80) | 1.17 (0.312) |
|  | 45-49 | 283 | 6.74 (6.74-6.75) | 149 | 6.99 (6.98-6.99) | 134 | 6.49 (6.49-6.50) | 0.93 (0.539) |
|  | 50-54 | 669 | 15.43 (15.42-15.43) | 340 | 15.54 (15.54-15.55) | 329 | 15.31 (15.31-15.32) | 0.99 (0.847) |
|  | 55-59 | 1075 | 31.16 (31.16-31.17) | 509 | 29.57 (29.56-29.58) | 566 | 32.75 (32.74-32.76) | 1.11 (0.094) |
|  | 60-64 | 1521 | 62.77 (62.76-62.78) | 692 | 58.31 (58.29-58.32) | 829 | 67.05 (67.04-67.07) | 1.15 (0.007) |
|  | 65-69 | 2280 | 118.74 (118.72-118.75) | 1084 | 119.38 (119.36-119.40) | 1196 | 118.16 (118.14-118.18) | 0.99 (0.806) |
|  | 70-74 | 4335 | 244.60 (244.57-244.62) | 1813 | 232.72 (232.69-232.76) | 2522 | 253.91 (253.88-253.94) | 1.09 (0.005) |
|  | 75-79 | 4216 | 344.94 (344.91-344.97) | 1657 | 345.51 (345.46-345.57) | 2559 | 344.57 (344.53-344.61) | 1.00 (0.931) |
|  | 80- | 3274 | 293.43 (293.40-293.46) | 1251 | 382.12 (382.05-382.19) | 2023 | 256.60 (256.57-256.64) | 0.67 (<0.001) |
|  | Total | 17977 | 35.56 (35.56-35.56) | 7659 | 30.29 (30.29-30.30) | 10318 | 40.82 (40.82-40.82) | 1.35 (<0.001) |
| 2014 | 0-19 | 13 | 0.12 (0.12-0.12) | 4 | 0.07 (0.07-0.07) | 9 | 0.18 (0.18-0.18) | 2.44 (0.126) |
|  | 20-24 | 13 | 0.38 (0.38-0.38) | 4 | 0.22 (0.22-0.22) | 9 | 0.56 (0.56-0.56) | 2.54 (0.108) |
|  | 25-29 | 16 | 0.51 (0.51-0.51) | 12 | 0.73 (0.73-0.74) | 4 | 0.26 (0.26-0.26) | 0.36 (0.064) |
|  | 30-34 | 47 | 1.18 (1.18-1.18) | 28 | 1.38 (1.38-1.38) | 19 | 0.98 (0.98-0.98) | 0.71 (0.244) |
|  | 35-39 | 68 | 1.75 (1.75-1.76) | 40 | 2.03 (2.03-2.03) | 28 | 1.47 (1.47-1.47) | 0.73 (0.191) |
|  | 40-44 | 154 | 3.42 (3.41-3.42) | 85 | 3.70 (3.70-3.71) | 69 | 3.12 (3.11-3.12) | 0.84 (0.287) |
|  | 45-49 | 317 | 7.37 (7.37-7.37) | 159 | 7.30 (7.29-7.30) | 158 | 7.44 (7.44-7.45) | 1.02 (0.859) |
|  | 50-54 | 655 | 15.15 (15.15-15.16) | 341 | 15.61 (15.60-15.61) | 314 | 14.69 (14.69-14.70) | 0.94 (0.438) |
|  | 55-59 | 1048 | 28.49 (28.49-28.50) | 551 | 30.01 (30.00-30.02) | 497 | 26.98 (26.97-26.99) | 0.90 (0.085) |
|  | 60-64 | 1542 | 61.16 (61.15-61.17) | 738 | 59.70 (59.68-59.71) | 804 | 62.57 (62.56-62.59) | 1.05 (0.356) |
|  | 65-69 | 2358 | 117.38 (117.37-117.40) | 1179 | 123.06 (123.04-123.09) | 1179 | 112.21 (112.19-112.23) | 0.91 (0.025) |
|  | 70-74 | 4009 | 224.58 (224.56-224.61) | 1746 | 221.18 (221.15-221.21) | 2263 | 227.28 (227.25-227.31) | 1.03 (0.393) |
|  | 75-79 | 4255 | 328.23 (328.20-328.26) | 1693 | 328.08 (328.03-328.13) | 2562 | 328.34 (328.30-328.38) | 1.00 (0.980) |
|  | 80- | 3613 | 299.40 (299.37-299.44) | 1433 | 398.28 (398.22-398.35) | 2180 | 257.40 (257.36-257.43) | 0.65 (<0.001) |
|  | Total | 18108 | 35.67 (35.67-35.67) | 8013 | 31.58 (31.58-31.58) | 10095 | 39.76 (39.76-39.76) | 1.26 (<0.001) |
| 2015 | 0-19 | 8 | 0.08 (0.08-0.08) | 3 | 0.06 (0.06-0.06) | 5 | 0.10 (0.10-0.10) | 1.80 (0.416) |
|  | 20-24 | 15 | 0.43 (0.43-0.43) | 8 | 0.43 (0.43-0.43) | 7 | 0.42 (0.42-0.43) | 0.99 (0.984) |
|  | 25-29 | 21 | 0.67 (0.67-0.67) | 13 | 0.80 (0.80-0.80) | 8 | 0.53 (0.53-0.54) | 0.67 (0.368) |
|  | 30-34 | 34 | 0.90 (0.90-0.90) | 19 | 0.98 (0.98-0.98) | 15 | 0.81 (0.81-0.81) | 0.83 (0.583) |
|  | 35-39 | 80 | 2.06 (2.06-2.06) | 41 | 2.08 (2.08-2.08) | 39 | 2.05 (2.04-2.05) | 0.98 (0.946) |
|  | 40-44 | 150 | 3.40 (3.40-3.40) | 88 | 3.92 (3.92-3.92) | 62 | 2.86 (2.86-2.87) | 0.73 (0.057) |
|  | 45-49 | 317 | 7.25 (7.25-7.25) | 171 | 7.72 (7.72-7.73) | 146 | 6.76 (6.76-6.77) | 0.88 (0.240) |
|  | 50-54 | 595 | 13.95 (13.94-13.95) | 332 | 15.37 (15.37-15.38) | 263 | 12.49 (12.48-12.49) | 0.81 (0.012) |
|  | 55-59 | 1099 | 28.31 (28.30-28.31) | 576 | 29.73 (29.72-29.74) | 523 | 26.89 (26.88-26.89) | 0.90 (0.096) |
|  | 60-64 | 1456 | 53.63 (53.62-53.63) | 700 | 52.56 (52.55-52.58) | 756 | 54.65 (54.63-54.66) | 1.04 (0.459) |
|  | 65-69 | 2143 | 101.59 (101.58-101.60) | 1036 | 102.48 (102.46-102.50) | 1107 | 100.77 (100.75-100.78) | 0.98 (0.695) |
|  | 70-74 | 3590 | 201.99 (201.97-202.01) | 1580 | 199.50 (199.47-199.53) | 2010 | 203.99 (203.96-204.01) | 1.02 (0.508) |
|  | 75-79 | 3899 | 288.04 (288.01-288.07) | 1603 | 293.68 (293.63-293.72) | 2296 | 284.23 (284.2-284.27) | 0.97 (0.314) |
|  | 80- | 3534 | 269.34 (269.32-269.37) | 1383 | 347.10 (347.04-347.15) | 2151 | 235.43 (235.4-235.47) | 0.68 (<0.001) |
|  | Total | 16941 | 33.25 (33.25-33.25) | 7553 | 29.67 (29.67-29.67) | 9388 | 36.82 (36.82-36.83) | 1.24 (<0.001) |
| * The incidence is reported as per 100,000 person-years for patients with Parkinson’s disease. | | | | | | | | |

eTable 5. Frequencies and incidence of drug-induced parkinsonism in Korea from 2012 to 2015

| **Calendar year** | **Age groups**  **(years)** | **Total** | | **Male** | | **Female** | | **Female-to-male ratio**  **(*P* value)** |
| --- | --- | --- | --- | --- | --- | --- | --- | --- |
|  |  | No. | Incidence  (95% CI) | No. | Incidence  (95% CI) | No. | Incidence  (95% CI) |  |
| 2012 | 0-19 | 138 | 1.23 (1.23-1.23) | 85 | 1.45 (1.45-1.45) | 53 | 0.99 (0.99-0.99) | 0.68 (0.028) |
|  | 20-24 | 142 | 4.37 (4.36-4.37) | 85 | 4.96 (4.96-4.96) | 57 | 3.70 (3.70-3.71) | 0.75 (0.087) |
|  | 25-29 | 171 | 5.04 (5.04-5.05) | 96 | 5.49 (5.49-5.50) | 75 | 4.57 (4.56-4.57) | 0.83 (0.231) |
|  | 30-34 | 219 | 5.46 (5.46-5.46) | 108 | 5.27 (5.27-5.28) | 111 | 5.66 (5.65-5.66) | 1.07 (0.604) |
|  | 35-39 | 375 | 9.10 (9.09-9.10) | 197 | 9.40 (9.40-9.41) | 178 | 8.78 (8.77-8.78) | 0.93 (0.507) |
|  | 40-44 | 459 | 10.05 (10.05-10.06) | 228 | 9.84 (9.84-9.84) | 231 | 10.27 (10.27-10.28) | 1.04 (0.646) |
|  | 45-49 | 423 | 10.20 (10.20-10.20) | 220 | 10.37 (10.37-10.38) | 203 | 10.02 (10.01-10.02) | 0.97 (0.720) |
|  | 50-54 | 432 | 10.07 (10.07-10.07) | 188 | 8.70 (8.70-8.71) | 244 | 11.46 (11.46-11.47) | 1.32 (0.004) |
|  | 55-59 | 302 | 9.30 (9.29-9.30) | 117 | 7.24 (7.24-7.24) | 185 | 11.34 (11.33-11.34) | 1.57 (<0.001) |
|  | 60-64 | 206 | 8.80 (8.80-8.80) | 90 | 7.85 (7.85-7.86) | 116 | 9.71 (9.70-9.72) | 1.24 (0.130) |
|  | 65-69 | 204 | 10.87 (10.86-10.87) | 72 | 8.19 (8.18-8.19) | 132 | 13.23 (13.23-13.24) | 1.62 (0.001) |
|  | 70-74 | 202 | 11.94 (11.93-11.94) | 60 | 8.13 (8.12-8.14) | 142 | 14.88 (14.87-14.89) | 1.83 (<0.001) |
|  | 75-79 | 190 | 16.48 (16.47-16.48) | 55 | 12.35 (12.34-12.36) | 135 | 19.07 (19.06-19.08) | 1.54 (0.006) |
|  | 80- | 106 | 10.22 (10.21-10.23) | 23 | 7.65 (7.64-7.66) | 83 | 11.27 (11.26-11.27) | 1.47 (0.099) |
|  | Total | 3569 | 7.09 (7.09-7.09) | 1624 | 6.45 (6.45-6.45) | 1945 | 7.73 (7.73-7.73) | 1.20 (<0.001) |
| 2013 | 0-19 | 131 | 1.19 (1.19-1.20) | 80 | 1.40 (1.40-1.40) | 51 | 0.97 (0.97-0.97) | 0.69 (0.040) |
|  | 20-24 | 121 | 3.61 (3.61-3.62) | 61 | 3.44 (3.44-3.45) | 60 | 3.80 (3.80-3.80) | 1.10 (0.588) |
|  | 25-29 | 158 | 4.90 (4.89-4.90) | 80 | 4.80 (4.79-4.80) | 78 | 5.00 (5.00-5.01) | 1.04 (0.790) |
|  | 30-34 | 211 | 5.22 (5.22-5.22) | 104 | 5.04 (5.04-5.04) | 107 | 5.41 (5.41-5.41) | 1.07 (0.606) |
|  | 35-39 | 306 | 7.69 (7.69-7.70) | 133 | 6.58 (6.58-6.58) | 173 | 8.85 (8.84-8.85) | 1.34 (0.010) |
|  | 40-44 | 457 | 10.02 (10.02-10.02) | 234 | 10.09 (10.08-10.09) | 223 | 9.95 (9.95-9.96) | 0.99 (0.888) |
|  | 45-49 | 428 | 10.20 (10.20-10.20) | 202 | 9.47 (9.47-9.48) | 226 | 10.95 (10.95-10.96) | 1.16 (0.133) |
|  | 50-54 | 363 | 8.37 (8.37-8.37) | 162 | 7.40 (7.40-7.41) | 201 | 9.35 (9.35-9.36) | 1.26 (0.027) |
|  | 55-59 | 275 | 7.97 (7.97-7.97) | 102 | 5.93 (5.92-5.93) | 173 | 10.01 (10.00-10.01) | 1.69 (<0.001) |
|  | 60-64 | 206 | 8.50 (8.50-8.51) | 74 | 6.24 (6.23-6.24) | 132 | 10.68 (10.67-10.68) | 1.71 (<0.001) |
|  | 65-69 | 170 | 8.85 (8.85-8.86) | 61 | 6.72 (6.71-6.72) | 109 | 10.77 (10.76-10.77) | 1.60 (0.003) |
|  | 70-74 | 213 | 12.02 (12.01-12.02) | 61 | 7.83 (7.82-7.84) | 152 | 15.30 (15.30-15.31) | 1.95 (<0.001) |
|  | 75-79 | 155 | 12.68 (12.68-12.69) | 52 | 10.84 (10.83-10.85) | 103 | 13.87 (13.86-13.88) | 1.28 (0.147) |
|  | 80- | 142 | 12.73 (12.72-12.73) | 42 | 12.83 (12.82-12.84) | 100 | 12.68 (12.68-12.69) | 0.99 (0.951) |
|  | Total | 3336 | 6.60 (6.60-6.60) | 1448 | 5.73 (5.73-5.73) | 1888 | 7.47 (7.47-7.47) | 1.30 (<0.001) |
| 2014 | 0-19 | 173 | 1.62 (1.62-1.62) | 119 | 2.14 (2.14-2.14) | 54 | 1.05 (1.05-1.05) | 0.49 (<0.001) |
|  | 20-24 | 156 | 4.54 (4.54-4.55) | 93 | 5.11 (5.10-5.11) | 63 | 3.91 (3.90-3.91) | 0.76 (0.099) |
|  | 25-29 | 202 | 6.41 (6.41-6.41) | 99 | 6.06 (6.05-6.06) | 103 | 6.79 (6.79-6.79) | 1.12 (0.417) |
|  | 30-34 | 268 | 6.75 (6.74-6.75) | 146 | 7.20 (7.19-7.20) | 122 | 6.28 (6.27-6.28) | 0.87 (0.265) |
|  | 35-39 | 341 | 8.80 (8.80-8.80) | 188 | 9.53 (9.53-9.54) | 153 | 8.04 (8.04-8.04) | 0.84 (0.118) |
|  | 40-44 | 430 | 9.54 (9.53-9.54) | 226 | 9.85 (9.84-9.85) | 204 | 9.21 (9.21-9.22) | 0.94 (0.493) |
|  | 45-49 | 431 | 10.02 (10.02-10.02) | 206 | 9.45 (9.45-9.46) | 225 | 10.60 (10.60-10.61) | 1.12 (0.235) |
|  | 50-54 | 400 | 9.25 (9.25-9.26) | 158 | 7.23 (7.23-7.24) | 242 | 11.32 (11.32-11.33) | 1.57 (<0.001) |
|  | 55-59 | 309 | 8.40 (8.40-8.40) | 112 | 6.10 (6.10-6.10) | 197 | 10.69 (10.69-10.70) | 1.75 (0.001) |
|  | 60-64 | 219 | 8.69 (8.68-8.69) | 80 | 6.47 (6.47-6.48) | 139 | 10.82 (10.81-10.82) | 1.67 (<0.001) |
|  | 65-69 | 181 | 9.01 (9.01-9.01) | 63 | 6.58 (6.57-6.58) | 118 | 11.23 (11.22-11.24) | 1.71 (0.001) |
|  | 70-74 | 239 | 13.39 (13.38-13.39) | 65 | 8.23 (8.23-8.24) | 174 | 17.48 (17.47-17.48) | 2.12 (<0.001) |
|  | 75-79 | 178 | 13.73 (13.72-13.74) | 56 | 10.85 (10.84-10.86) | 122 | 15.64 (15.63-15.64) | 1.44 (0.023) |
|  | 80- | 138 | 11.44 (11.43-11.44) | 36 | 10.01 (10.00-10.02) | 102 | 12.04 (12.04-12.05) | 1.20 (0.338) |
|  | Total | 3665 | 7.22 (7.22-7.22) | 1647 | 6.49 (6.49-6.49) | 2018 | 7.95 (7.95-7.95) | 1.22 (<0.001) |
| 2015 | 0-19 | 345 | 3.31 (3.31-3.31) | 228 | 4.21 (4.21-4.21) | 117 | 2.33 (2.33-2.33) | 0.55 (<0.001) |
|  | 20-24 | 342 | 9.75 (9.74-9.75) | 172 | 9.24 (9.23-9.24) | 170 | 10.32 (10.32-10.33) | 1.12 (0.304) |
|  | 25-29 | 326 | 10.41 (10.41-10.42) | 183 | 11.22 (11.22-11.23) | 143 | 9.53 (9.53-9.54) | 0.85 (0.143) |
|  | 30-34 | 448 | 11.80 (11.80-11.80) | 214 | 11.01 (11.01-11.02) | 234 | 12.62 (12.62-12.63) | 1.15 (0.149) |
|  | 35-39 | 614 | 15.82 (15.82-15.82) | 306 | 15.50 (15.49-15.50) | 308 | 16.15 (16.15-16.16) | 1.04 (0.609) |
|  | 40-44 | 894 | 20.27 (20.27-20.27) | 465 | 20.71 (20.71-20.72) | 429 | 19.81 (19.80-19.82) | 0.96 (0.505) |
|  | 45-49 | 975 | 22.30 (22.29-22.30) | 481 | 21.72 (21.71-21.73) | 494 | 22.89 (22.88-22.89) | 1.05 (0.414) |
|  | 50-54 | 864 | 20.25 (20.25-20.26) | 415 | 19.21 (19.21-19.22) | 449 | 21.32 (21.31-21.32) | 1.11 (0.127) |
|  | 55-59 | 754 | 19.42 (19.42-19.42) | 368 | 18.99 (18.99-19.00) | 386 | 19.84 (19.84-19.85) | 1.04 (0.548) |
|  | 60-64 | 456 | 16.79 (16.79-16.80) | 190 | 14.27 (14.26-14.27) | 266 | 19.23 (19.22-19.23) | 1.35 (0.002) |
|  | 65-69 | 310 | 14.70 (14.69-14.70) | 129 | 12.76 (12.75-12.77) | 181 | 16.48 (16.47-16.48) | 1.29 (0.026) |
|  | 70-74 | 327 | 18.40 (18.39-18.40) | 105 | 13.26 (13.25-13.27) | 222 | 22.53 (22.52-22.54) | 1.70 (<0.001) |
|  | 75-79 | 253 | 18.69 (18.68-18.70) | 63 | 11.54 (11.53-11.55) | 190 | 23.52 (23.51-23.53) | 2.04 (<0.001) |
|  | 80- | 150 | 11.43 (11.43-11.44) | 42 | 10.54 (10.53-10.55) | 108 | 11.82 (11.81-11.83) | 1.12 (0.528) |
|  | Total | 7058 | 13.85 (13.85-13.85) | 3361 | 13.20 (13.20-13.20) | 3697 | 14.50 (14.50-14.50) | 1.10 (<0.001) |
| * The incidence is reported as per 100,000 person-years for patients with drug-induced parkinsonism. | | | | | | | | |
