# Supplementary material for: Prevalence and incidence of Parkinson’s disease and drug-induced parkinsonism in Korea
Source: BMC Public Health. 2019 Oct 22;19:1328. doi: 10.1186/s12889-019-7664-6 (PMC6805681; doi:10.1186/s12889-019-7664-6)
Supplement: Supplementary file 2 — Additional file 2: Figure S1. Sensitivity analysis. Incidence of drug-induced parkinsonism in Korea. [file 12889_2019_7664_MOESM2_ESM.docx]

| A | Incidence, total^1)^ |  |  |
| --- | --- | --- | --- |
|  | |  | |
| B | Incidence, male^2)^ | C | Incidence, female^2)^ |
|  | |  | |
| DIP, drug-induced parkinsonism.  1) The average incidence per 100,000 person-years of DIP (A) between 2012-2015 in Korea.  2) The incidence per 100,000 person-years of DIP (B and C) by age group, sex and calendar year from 2012 to 2015 in Korea. | | | |

**Supplement eFigure 1. Sensitivity analysis. Incidence of drug-induced parkinsonism in Korea**
