# Supplementary material for: Prevalence and incidence of Parkinson’s disease and drug-induced parkinsonism in Korea
Source: BMC Public Health. 2019 Oct 22;19:1328. doi: 10.1186/s12889-019-7664-6 (PMC6805681; doi:10.1186/s12889-019-7664-6)
Supplement: Supplementary file 3 — Additional file 3: Figure S2. Sensitivity analysis. Number of incident patients with drug-induced parkinsonism by the types and numbers of offending drugs in Korea [file 12889_2019_7664_MOESM3_ESM.docx]

| A | The types of offending drugs |
| --- | --- |
|  | |
| B | The numbers of offending drugs’ use |
|  | |
| DIP, drug-induced parkinsonism.  The types of offending drugs (A) and the numbers of offending drugs’ use (B) were identified on the day of DIP occurrence from 2012 to 2015 in Korea. | |

**Supplement eFigure 2. Sensitivity analysis. Number of incident patients with drug-induced parkinsonism by the types and numbers of offending drugs in Korea**
